# Biapenem, a Carbapenem Antibiotic, Elicits Mycobacteria Specific Immune Responses and Reduces the Recurrence of Tuberculosis

**DOI:** 10.1128/spectrum.00858-23

**Published:** 2023-06-05

**Authors:** Isha Pahuja, Akanksha Verma, Antara Ghoshal, Suparba Mukhopadhyay, Anjna Kumari, Aishwarya Shaji, Shivam Chaturvedi, Ved Prakash Dwivedi, Ashima Bhaskar

**Affiliations:** a Immunobiology Group, International Centre for Genetic Engineering and Biotechnology, New Delhi, India; b Department of Molecular Medicine, Jamia Hamdard University, New Delhi, India; Griffith University

**Keywords:** tuberculosis, immunotherapy, Biapenem, memory T-cells, reactivation, reinfection, antimicrobials, *Mycobacterium tuberculosis*, multidrug resistance

## Abstract

Tuberculosis (TB) still tops the list of global health burdens even after COVID-19. However, it will sooner transcend the current pandemic due to the prevailing risk of reactivation of latent TB in immunocompromised individuals. The indiscriminate misuse and overuse of antibiotics have resulted in the emergence of deadly drug-resistant variants of Mycobacterium tuberculosis (*M.tb*). This study aims to characterize the functionality of the carbapenem antibiotic-Biapenem (BPM) in generating long-lasting immunity against TB. BPM treatment significantly boosted the activation status of the innate immune arm-macrophages by augmenting p38 signaling. Macrophages further primed and activated the adaptive immune cells CD4^+^ and CD8^+^ T-cells in the lung and spleen of the infected mice model. Furthermore, BPM treatment significantly amplified the polarization of T lymphocytes toward inflammatory subsets, such as Th1 and Th17. The treatment also helped generate a long-lived central memory T-cell subset. The generation of central memory T lymphocyte subset upon BPM treatment in the murine model led to a significant curtailing in the recurrence of TB due to reactivation and reinfection. These results suggest the potentiality of BPM as a potent adjunct immunomodulator to improve host defense against *M.tb* by enriching long-term protective memory cells.

**IMPORTANCE** Tuberculosis (TB) caused by Mycobacterium tuberculosis (*M.tb*) tops the list of infectious killers around the globe. The emergence of drug-resistant variants of *M.tb* has been a major hindrance toward realizing the “END TB” goal. Drug resistance has amplified the global burden toward the quest for novel drug molecules targeting *M.tb*. Host-directed therapy (HDT) offers a lucrative alternative to tackle emerging drug resistance and disease relapse by strengthening the host’s immunity. Through our present study, we have tried to characterize the functionality of the carbapenem antibiotic-Biapenem (BPM). BPM treatment significantly augmented long-lasting immunity against TB by boosting the innate and adaptive immune arms. The generation of long-lived central memory T lymphocyte subset significantly improved the disease outcome and provided sterilizing immunity in the murine model of TB. The present investigation's encouraging results have helped us depict BPM as a potent adjunct immunomodulator for treating TB.

## INTRODUCTION

Tuberculosis (TB) is an age-old disease caused by Mycobacterium tuberculosis (*M.tb*) that still affects a majority of the population worldwide. TB was the leading cause of death by a single infectious agent until the COVID-19 pandemic (1, 2). The incidence of the pandemic has reversed the progress in TB diagnosis and treatment to date and further aggravated the global TB burden (3).

The treatment of TB comprises a first line anti-tubercular therapy (ATT) with directly observed treatment short course (DOTS), a customary prescribed by the WHO as a TB control measure, and it consists of 4 drugs, Isoniazid, Rifampicin, Pyrazinamide, and Ethambutol. The treatment course duration runs from 6 to 8 months (4). From the duration of therapy being too prolonged that adversely affects the financial burden in the lower-income strata countries where the prevalence of TB has been very prominent, the subsequent chance of relapse of the disease by reinfection even after the cure post treatment, may cause immune dampening in the patients, meaning DOTS does not pose as a promising therapy to TB. Furthermore, the cost and duration of treatment, and the lack of proper infrastructure, often enforce the patients to stop it midway, consequently negatively impacting them and leading to drug resistance (5). The emergence of drug resistance in TB dampened the progress made in curbing the disease even more. The success of multi-drug resistant (MDR) and extensively drug resistant (XDR) tuberculosis treatments cater the use of fluoroquinolones as first line drugs and also depend on the degree of the resistance. The length of treatment for MDR and XDR TB is not defined and varies from patient to patient (6).

The infidelity of the progression of the disease also caters to the difficulty in its prophylactic measures too. The Bacille Calmette-Guerin (BCG) vaccine is the only approved chemoprophylactic measure against TB infection to date. Its efficacy is most pronounced in disseminated forms of TB in children compared to pulmonary TB in adults (7).

Beta-lactam antibiotics, broad-spectrum drugs that have been used to treat many Gram-negative bacterial infections by inhibiting the cell wall synthesis of bacteria and carve a niche in treatment against the drug-resistant bacteria. Conventionally, these beta-lactam antibiotics were not used against treating TB pertaining to the presence of an extremely active β-lactamase (BlaC) that is chromosomally encoded in *M.tb* (8). However, carbapenems, a subclass of beta-lactam antibiotics, have a unique structural composition that makes them non-susceptible to the β-lactamase activity of *M.tb* and hence a poor substrate for BlaC. This property of the carbapenems made them an efficient target for treatment against drug-resistant *M.tb* strains (8, 9). Biapenem (BPM), one of the most common commercially available carbapenems, has a potent efficacy as anti-tuberculous agent against drug-susceptible and drug-resistant *M.tb* strains compared to other carbapenems owing to its robust pharmacokinetic activities (10, 11). Recent studies show BPM exhibiting antibacterial activity and antimicrobial activity against *M.tb* in both *in vitro* and *in vivo* settings. BPM also exhibits synergy with Rifampicin, one of the first line anti-TB drug against drug-susceptible *M.tb* infections (11–13). Since most of the current ATT drugs cause severe immune dampening (14), effects of BPM on the host immune responses should be explored to project BPM as a potential candidate for ATT.

Here, we have examined the immunomodulatory actions of -BPM during *M.tb* infection *ex vivo* and *in vivo*. Our results demonstrate the efficacy of -BPM as a potent modulator of innate and adaptive immune responses. BPM regulates various host defense mechanisms aiding in combating *M.tb* infection. BPM boosted the antimicrobial activity of the macrophages by regulating ROS levels and p38 MAPK signaling. It also enhanced the expression of pro-inflammatory cytokines and co-stimulatory molecules. The activated innate immune responses upon BPM treatment played a vital role for further stimulation of the adaptive immune responses. With enhanced T cell activation, BPM treatment significantly enriched the protective T cell responses in the lungs and the spleen of *M.tb* infected mice. Moreover, BPM treatment enhanced the expression of IFN-γ and IL-17, the proinflammatory cytokines known to play a significant role in rendering protection to the host against the bacterial infection. Further immune analysis revealed enhanced T-cell memory responses in the BPM treated mice. It has been established that the central memory (T_CM_) and effector memory (T_EM_) T-cells play a vital role in the protection of host during recall responses (15–17). One of the major challenges associated with TB prophylaxis is the persistent recurrence of the disease in the form of reactivation or reinfection; our study demonstrated that BPM plays a substantial role in reduction of this recurrence. Considering all these beneficial facets of BPM against *M.tb*, it can potentiate as an immunomodulator against TB pathogenesis and can translate to prospective aid in treatment to human patients as well.

## RESULTS

### Biapenem enhances antimycobacterial potential of macrophages by modulating P38 signaling pathway.

**FIG 1:**
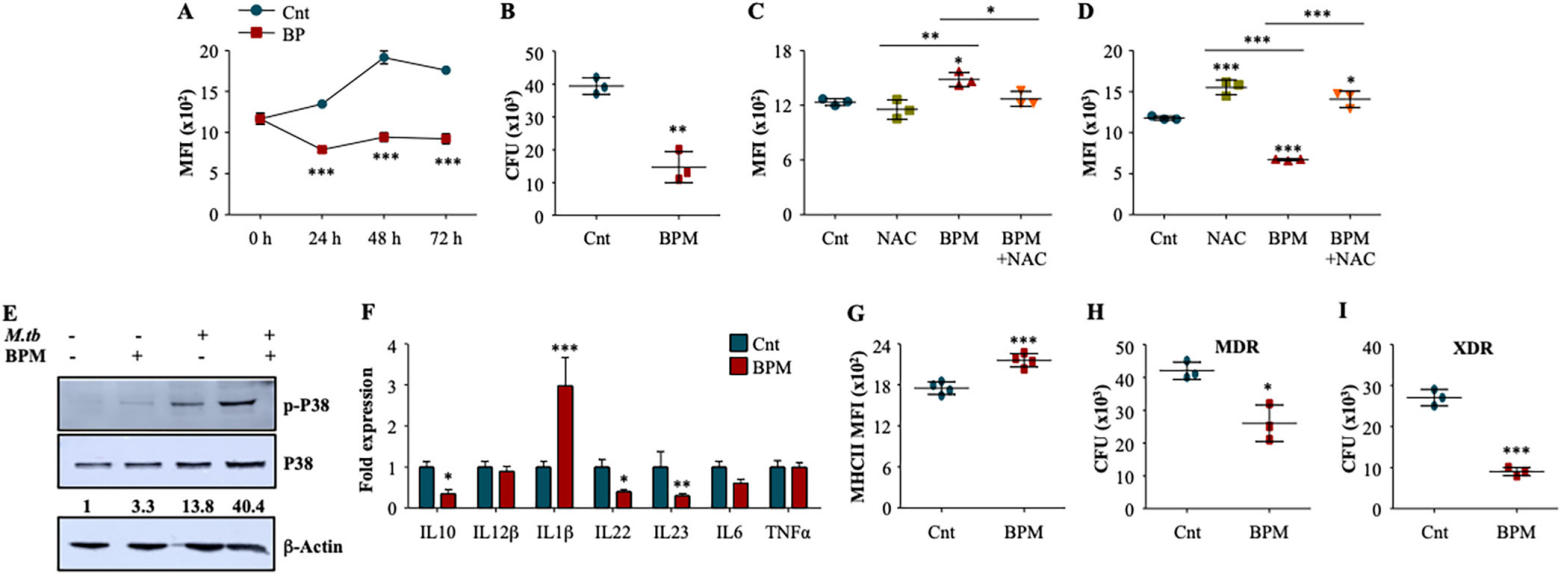
Biapenem augments the anti-mycobacterial activity of macrophages by modulating p38 MAPK signaling. Murine macrophages were infected with GFP expressing *M.tb* H37Rv at MOI 1:1 and treated with BPM (10 μg/mL). (A) Intracellular GFP fluorescence at different time points postinfection with and without BPM treatment. (B) Peritoneal macrophages were infected with *M.tb* H37Rv at MOI 1:10 followed by treatment with BPM (10 μg/mL) for 48 h. CFU determination at 48h post treatment. (C) and (D) Mouse peritoneal macrophages pretreated with 10 mM NAC were infected with GFP expressing *M.tb* H37Rv at an MOI of 1:1 followed by treated with 10 μg/mL BPM. (C) Intracellular ROS levels 48 h postinfection. (D) Corresponding bacterial survival post 48 h BPM and NAC treatment. (E) Western blots analysis to show the phosphorylation of P38 in uninfected and infected mouse peritoneal macrophages with or without BPM treatment for 2 h. (F) Fold change in the expression of cytokines in the infected macrophages with or without BPM treatment. (G) Expression of MHC-II on *M.tb* infected peritoneal macrophages 48 h post treatment with 10 μg/mL BPM. (H) and (I) Peritoneal macrophages were infected with *M.tb* Jal 2261 (MDR) and MYC 431 (XDR) at MOI 1:10, followed by treatment with BPM (10 μg/mL). (H) MDR and (I) XDR bacterial burden at 48 h post treatment. Data is representative of 3 independent experiments. The results shown are means ± SD (*n* = 3 or 4). *P* value * ≤ 0.05, ** ≤ 0.005, *** ≤ 0.0005.

BPM, a carbapenem antibiotic, has already been shown to have anti-mycobacterial potential in several studies (11, 17). It has been shown that BPM has synergistic antimycobacterial effect with rifampicin both *in vitro* and *in vivo* (12, 13). However, none of the studies explore its effect on the host immunity. To begin, we assessed the time kinetics of intra-macrophage bacterial growth in the presence of BPM. For this, mice peritoneal macrophages were infected with *M.tb* H37Rv-GFP (MOI 1:1) and treated with BPM followed by flow cytometry to determine the bacterial growth at different time points. We observed that BPM significantly induces the potential of macrophages to restrict the mycobacterial growth in time dependent manner (Fig. 1A). Further, CFU determination at 48 h postinfection with *M.tb* H37Rv (MOI 1:10) indicated similar results (Fig. 1B). Macrophages employ several defense mechanisms to restrict the growth of the pathogens, such as the generation of reactive oxygen species (ROS) and reactive nitrogen species (RNS) (18) .We observed that BPM treatment induced significant levels of ROS in the infected macrophages as observed by an increase in the CellROX fluorescence (Fig. 1C), which was reduced upon treatment with the antioxidant, N-acetylcysteine (NAC) (19). Furthermore, NAC co-treatment abolished the anti-mycobacterial potential of BPM indicating ROS generation as one of the major mechanisms by which BPM exerts its effect (Fig. 1D).

Since ROS is known to induce multiple intracellular signaling pathways (20–22), we analyzed the activation status of MAPK pathway upon BPM treatment. We observed that BPM activates p38 signaling (Fig. 1E), which further led to the induction of pro-inflammatory cytokines (Fig. 1F) that mediate bacterial killing and are also required for T cell differentiation toward the protective Th1 and Th17 cells (23–26). Further, BPM treatment led to enhanced expression of MHCII on the infected macrophages which may aid in T cell activation (Fig. 1G) (27, 28). Lastly, BPM displayed significant anti-tubercular activity against drug-resistant clinical isolates of *M.tb* (Fig. 1H and I). Collectively, our *ex vivo* data indicates that BPM treatment positively induces the activation of macrophages and stimulates the pro-inflammatory environment to mediate the host resistance to TB.

### Biapenem restricts the mycobacterial growth in mice.

**FIG 2:**
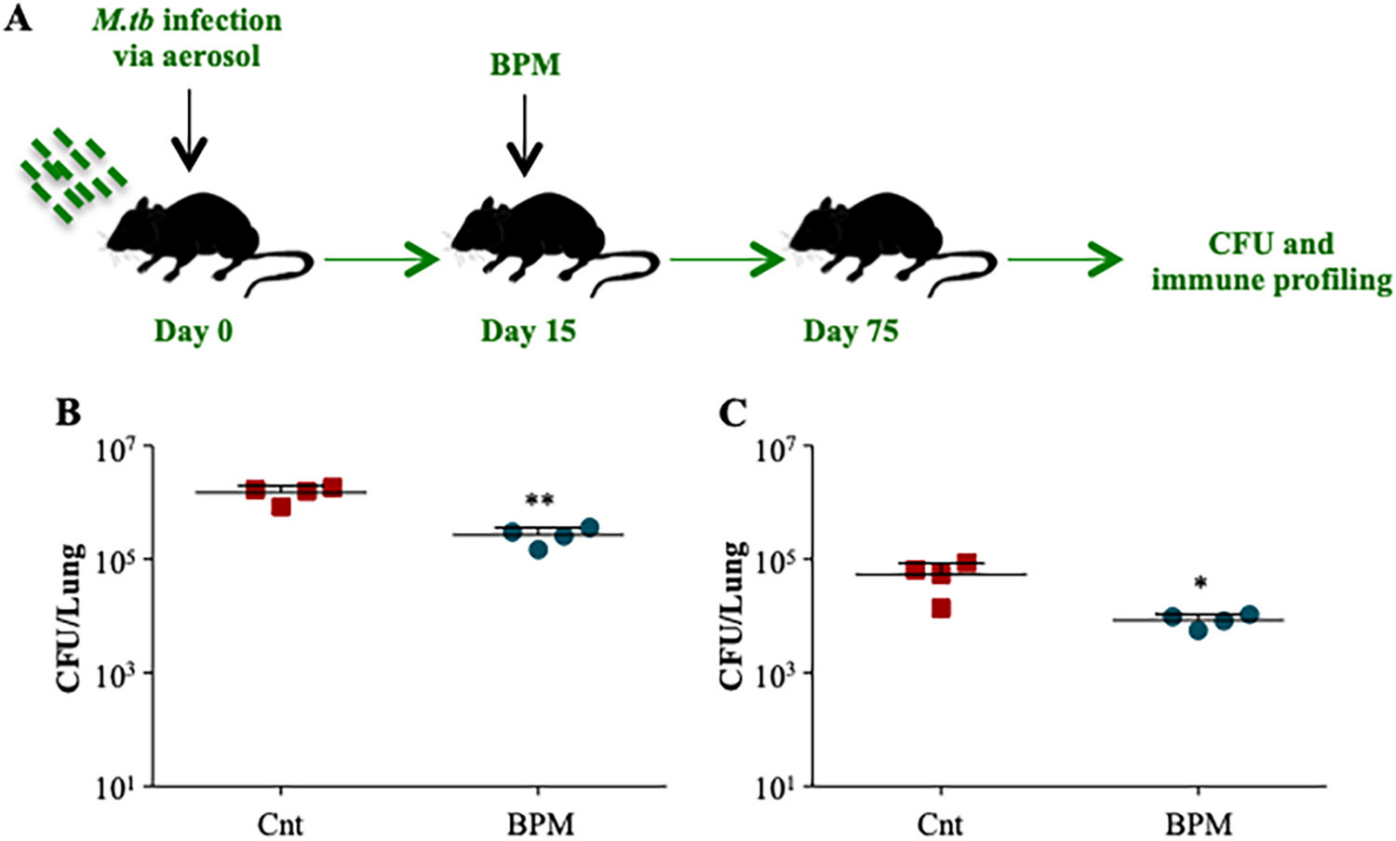
Biapenem treatment restricts the mycobacterial growth *in vivo*. (A) Schematic representation of mice model used in the study. Bacterial burden in the (B) lungs and (C) spleen of BPM treated animals 75 days postinfection. Data is representative of 2 independent experiments. The data values represent mean ± SD (*n* = 4). *, *P* < 0.05, **, *P* < 0.005, ***, *P* < 0.0005.

To corroborate our *ex vivo* findings, we performed an *in vivo* experiment wherein C57BL/6 mice were infected with *M.tb* H37Rv through low dose aerosol infection model. Group of animals were either kept untreated or were treated with BPM (5 mg/kg) for 60 days followed by determination of bacterial burden and immune profiling (Fig. 2A). BPM treatment significantly lowered the bacillary load in the lungs (Fig. 2B) and the spleen (Fig. 2C) of *M.tb* infected mice compared to the untreated control group as reported earlier.

### Biapenem activates innate immune cells during TB.

**FIG 3:**
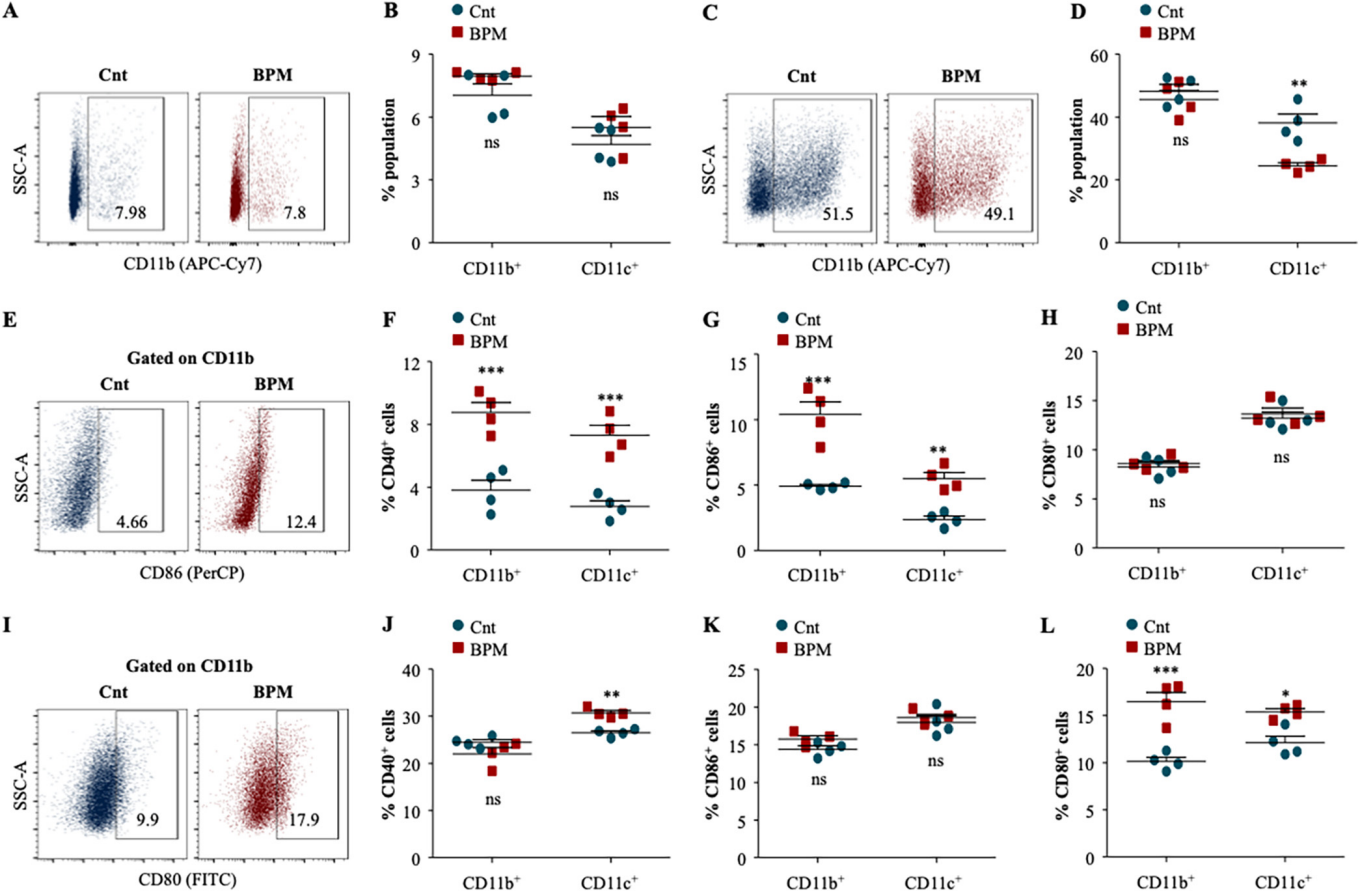
Biapenem treatment induces innate immune activation crucial for the activation of adaptive immunity in *in vivo* settings. Single cell suspensions generated from the infected lungs and spleen were *ex vivo* stimulated with *M.tb* complete soluble antigen (CSA) for overnight followed by surface staining with antibodies against CD11b (APCCy7), CD11c (APC), CD80 (FITC), CD86 (PerCPCy5.5), and CD40 (PE) and subjected to flow cytometry. (A) Representative dot plots and the percentage of (B) CD11b^+^ and CD11c^+^ cells in the infected and BPM treated spleen. (C) Representative dot plots depicting the percentage of (D) CD11b^+^ and CD11c^+^ cells in the infected and BPM treated lung. (E) Representative dot plots and the percentage of (F) CD40^+^, (G) CD86^+^, and (H) CD80^+^ cells in the CD11b_+_ and CD11c^+^ splenic cells. (I) Representative dot plots and the percentage of (J) CD11b^+^CD40^+^ and CD11c^+^CD40^+^ cells, (K) CD11b^+^CD86^+^ and CD11c^+^CD86^+^ cells, and (L) CD11b^+^CD80^+^ and CD11c^+^CD80^+^ cells in the lungs of infected and BPM treated animals. Data is representative of 2 independent experiments. The data values represent mean ± SD (*n* = 4). *, *P* < 0.05, **, *P* < 0.005, ***, *P* < 0.0005.

As observed in Fig. 1, BPM treatment modulated intracellular signaling to activate macrophages. To further evaluate the potential of BPM in activating innate immunity *in vivo*, macrophage activation was assessed in infected and BPM treated lungs and spleen. Gating strategy employed in this study is depicted in Fig. S1. While no significant increase in the CD11b^+^ and CD11c^+^ cells was observed in the spleen of infected animals (Fig. 3A and B), BPM significantly enhanced the percentage of CD11c^+^ cells in the infected lungs (Fig. 3C and D). Henceforth, the co-stimulatory and activation markers CD80, CD86, and CD40 were analyzed in the BPM treated infected lung and spleen to measure the activation of innate cells. There was a substantial increase in the expression of CD86 and CD40 on CD11b^+^ and CD11c^+^ cells upon BPM treatment in the infected spleen (Fig. 3E to G), while no significant increase in the expression of CD80 (Fig. 3H) was observed. Further, BPM treated innate cells in the lungs showed significant enhancement in the expression of CD80 and CD40 with no effect on CD86 (Fig. 3J and L). Altogether, this data suggests that BPM induces an innate immune response via the activation of macrophages and dendritic cells in the lungs and the spleen of infected mice, which is required to activate adaptive immunity.

### Biapenem induces long-lasting protective T cell immunity by enriching the pool of central memory T cells in mice.

**FIG 4:**
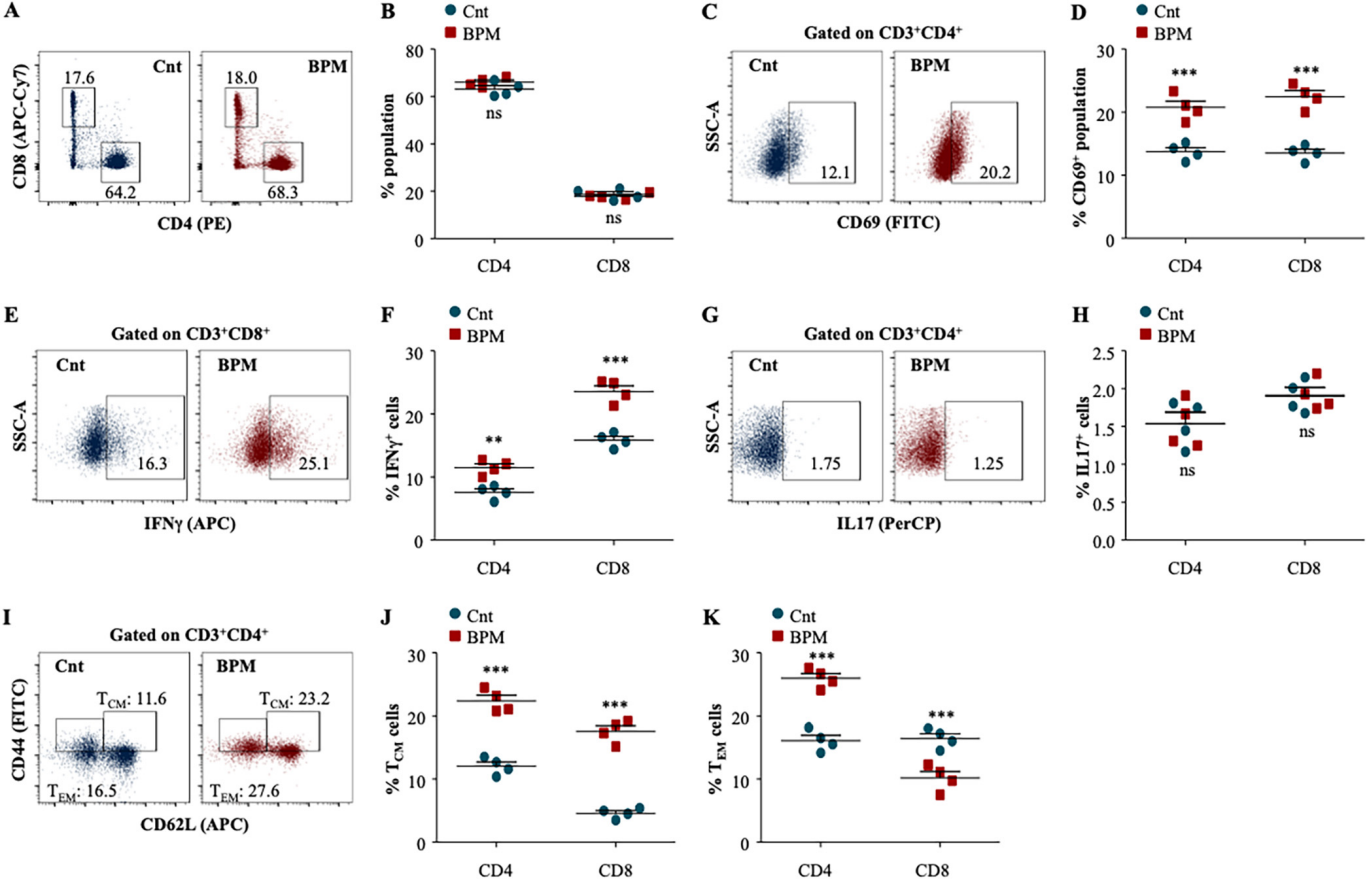
Biapenem treatment augments T cell responses required for long-term protection against tuberculosis in the spleen of infected mice. (A) to (D) *Ex vivo* stimulated splenocytes were surface stained with α-CD3 (Pacific Blue), α-CD4 (PE), α-CD8 (APCCy7), and α-CD69 (FITC) followed by flow cytometry. (A) Representative dot plots and (B) the percentage of CD4^+^ and CD8^+^ T cells in the infected spleen. (C) Representative dot plots and (D) the percentage of CD4^+^CD69^+^ and CD8^+^CD69^+^ T cells in the spleen of infected mice. (E) to (H) *Ex vivo* stimulated splenocytes were treated with monensin and brefeldin A for 2 h and surface stained with α-CD3 (Pacific Blue), α-CD4 (PE), and α-CD8 (APCCy7) followed by intracellular staining with α-IFNγ (APC) and α-IL-17 (PerCP). (E) Representative dot plots and (F) the percentage of CD4^+^IFNγ^+^ and CD8^+^IFNγ^+^ T cells in the spleen of infected and BPM treated mice. (F) Representative dot plots and (G) the percentage of CD4^+^IL-17^+^ and CD8^+^IL-17^+^ T cells in the spleen of infected and BPM treated mice. (I) to (K) *Ex vivo* stimulated splenocytes were surface stained with α-CD3 (Pacific Blue), α-CD4 (PE), α-CD8 (APCCy7), α-CD62L (APC), and α-CD44 (FITC) followed by flow cytometry. (I) Representative dot plots and (J) the percentage of CD4^+^/CD8^+^ T_CM_ cells (CD62L^HI^CD44^HI^) and (K) CD4^+^/CD8^+^ T_EM_ (CD62L^LO^CD44^LO^) cells in the infected and BPM treated spleen of the mice. Data is representative of 2 independent experiments. The data values represent mean ± SD (*n* = 4). *, *P* < 0.05, **, *P* < 0.005, ***, *P* < 0.0005.

Host immunity plays a critical role in restricting the dissemination of *M.tb* and therefore, only a small population develops active TB despite being latently infected. A critical balance of immune responses is required to contain the infection. It has been largely established that IFN-γ-producing CD4^+^ T cells provides protective immunity against TB and any defect in Th1 signaling leads to the unrestricted growth of *M.tb* (29, 30). Moreover, IL-4 producing Th2 cells assists in disease progression (31, 32). Recently, we and others have established that Th17 cells largely provide protection during secondary infection synergistically with Th1 cells (33–35). In the previous result, we established that BPM treatment significantly induces the pro-inflammatory signaling in macrophages, which are very critical to activate adaptive immunity. Therefore, we extended our interest in profiling the T cell responses after BPM treatment. With no change in the percentage of CD4^+^ and CD8^+^ T-cells (Fig. 4A and B), the expression of activation marker CD69 was significantly enhanced in the splenic CD4^+^ and CD8^+^ T cells (Fig. 4C and D).

Furthermore, BPM treatment significantly induced the IFN-γ producing T cell (Fig. 4E and F) with no change in the IL-17 producing T cell (Fig. 4G and H) responses in the spleen of infected animals.

Strong pro-inflammatory innate and adaptive immunity is not sufficient to achieve sterile immunity. Long-term sustained immune responses in terms of memory cells are critically required to control the infection as well as to reduce the host vulnerability to disease reinfection and reactivation (15, 16, 36). Upon antigen encounter, T naive cells differentiate into central memory T-cells (T_CM_) and effector memory T-cells (T_EM_). T_EM_ cells are short-lived, have limited proliferative capacity, and participate in the immediate elimination of the pathogens whereas, T_CM_ cells can effectively proliferate and further gives rise to T_EM_ cells to control the infection. We assessed the impact of BPM treatment on different T cell memory subsets within the CD4^+^ and CD8^+^ T cell population (Fig. 4I). We observed a significant increase in the percentage of CD4^+^ and CD8^+^ T_CM_ cells (Fig. 4J) in the spleen of infected animals. While BPM treatment increased CD4^+^ T_EM_ cells, CD8^+^ T_EM_ cells were decreased upon BPM treatment in the spleen of infected animals (Fig. 4K).

**FIG 5:**
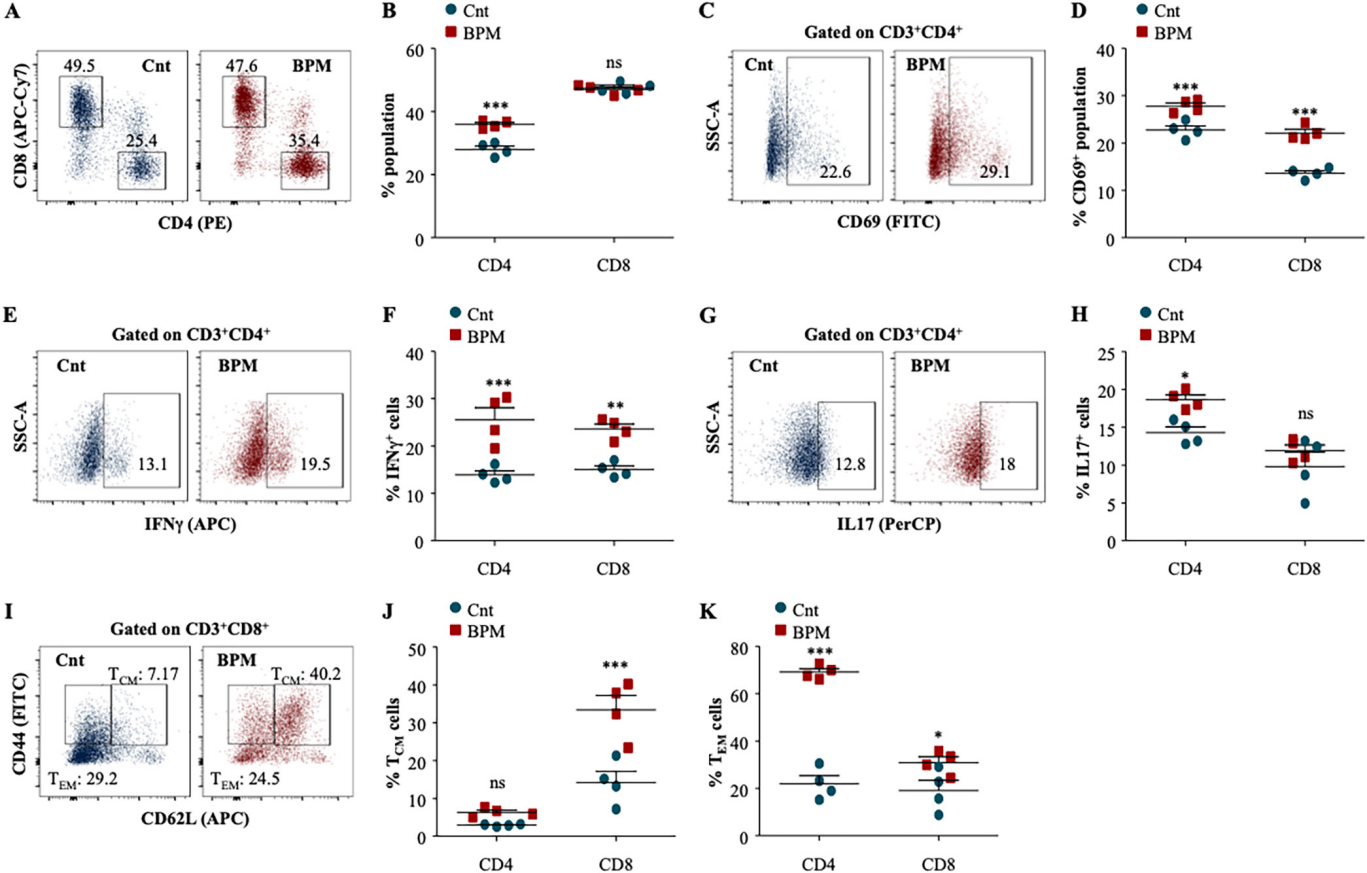
Biapenem treatment instigates long-term protective T cell immunity against tuberculosis in the lung of mice. (A) to (D) *Ex vivo* stimulated lung cells were surface stained with α-CD3 (Pacific Blue), α-CD4 (PE), α-CD8 (APCCy7), and α-CD69 (FITC) followed by flow cytometry. (A) Representative dot plots and (B) the percentage of CD4^+^ and CD8^+^ T cells in the infected lungs. (C) Representative dot plots and (D) the percentage of CD4^+^CD69^+^ and CD8^+^CD69^+^ T cells in the lungs of infected mice. (E) to (H) *Ex vivo* stimulated lung cells were treated with monensin and brefeldin A for 2 h and surface stained with α-CD3 (Pacific Blue), α-CD4 (PE), and α-CD8 (APCCy7) followed by intracellular staining with α-IFNγ (APC) and α-IL-17 (PerCP). (E) Representative dot plots and (F) the percentage of CD4^+^IFNγ^+^ and CD8^+^IFNγ^+^ T cells in the lungs of infected and BPM treated mice. (F) Representative dot plots and (G) the percentage of CD4^+^IL-17^+^ and CD8^+^IL-17^+^ T cells in the lungs of infected and BPM treated mice. (I) to (K) *Ex vivo* stimulated lungs were surface stained with α-CD3 (Pacific Blue), α-CD4 (PE), α-CD8 (APCCy7), α-CD62L (APC), and α-CD44 (FITC) followed by flow cytometry. (I) Representative dot plots and (J) the percentage of CD4^+^/CD8^+^ T_CM_ cells (CD62L^HI^CD44^HI^) and (K) CD4^+^/CD8^+^ T_EM_ (CD62L^LO^CD44^LO^) cells in the infected and BPM treated lungs. Data is representative of 2 independent experiments. The data values represent mean ± SD (*n* = 4). *, *P* < 0.05, **, *P* < 0.005, ***, *P* < 0.0005.

BPM treated and *M.tb* infected lungs were also analyzed T cell mediated immune responses. BPM treatment significantly increased the percentage of CD4^+^ T cells with no effect on the CD8^+^ T cells (Fig. 5A and B). However, treatment with BPM significantly enhanced the percentage activation of CD4^+^ and CD8^+^ T cells as evidenced by the expression of CD69 on the surface of CD4^+^ and CD8^+^ T cells in the lung of infected animals (Fig. 5C and D). Moreover, IFNγ producing CD4^+^ and CD8^+^ T cells and IL-17 producing CD4^+^ T cells were also enriched in the lung of the BPM treated animals (Fig. 5E to H). Furthermore, BPM treatment significantly induced the percentage of CD8^+^ T_CM_ with modest effect on CD4^+^ T_CM_ cells (Fig. 5I and K). However, there was significant increase in the percentage of CD4^+^T_EM_ and CD8^+^ T_EM_ in the lung of infected animals (Fig. 5L). Altogether, these results strongly suggest that BPM treatment strengthens the long-lasting protective immunity in the *M.tb* infected animals.

### Biapenem treatment reduces the extent of TB recurrence.

**FIG 6:**
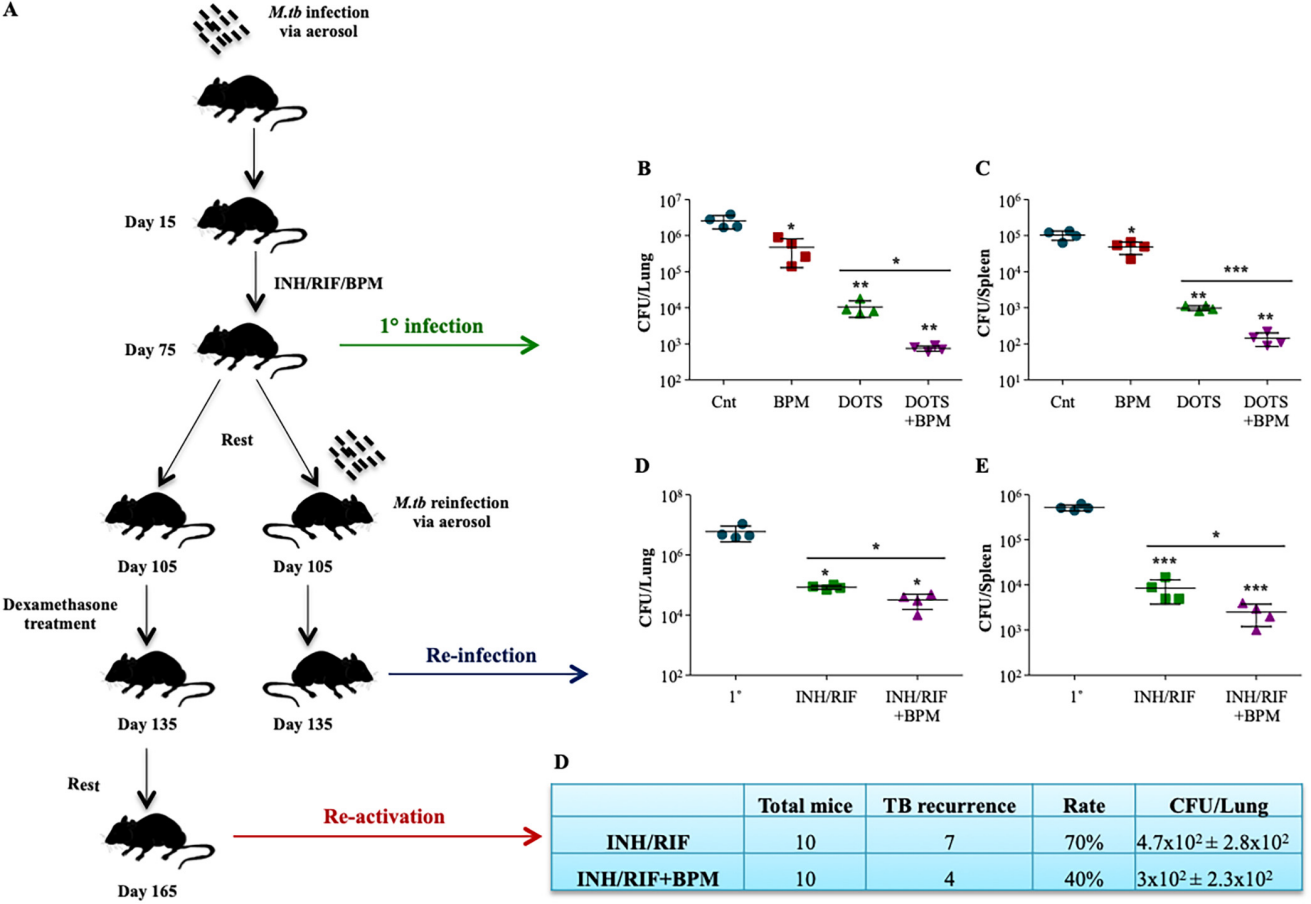
Biapenem reduces the recurrence of tuberculosis reinfection and reactivation. (A) Schematic representation of primary infection, re-infection, and re-activation mice model used in the study. Bacterial burden in the (B) lungs and (C) spleen of BPM and INH/RIF treated animals 75 days postinfection. Bacterial burden in the (D) lungs and (E) spleen of BPM and INH/RIF treated and reinfected animals. (F) Rate of disease relapse with and without BPM treatment in re-activation group. Data is representative of 2 independent experiments. The data values represent mean ± SD (*n* = 4 to 10). *, *P* < 0.05, **, *P* < 0.005, ***, *P* < 0.0005.

The current available antituberculosis therapy fails to provide 100% sterilization. Moreover, due to the severe immune dampening side effects of the therapy, the host is left vulnerable to disease reinfection and relapse (14, 37, 38). Central memory T cells play vital roles in providing long-lasting protection and reduces the host vulnerability to TB reactivation and reinfection. Since BPM treatment positively modulates the host immune responses against *M.tb* and enriches the central memory T cells in *M.tb* infected mice, we evaluated the potential of BPM to be an adjunct to the current treatment regime. The impact of BPM treatment against recurrent infection and re-activation of TB was also analyzed. C57BL/6 mice were infected with *M.tb* H37Rv through low dose aerosol infection model and after 15 days these mice were treated with either INH+RIF (Cnt group) or INH+RIF+BPM (treatment group) for the next 60 days, followed by CFU analysis (Fig. 6A). After 30 days rest, the mice were re-challenged with *M.tb* H37Rv through low dose aerosol infection model for reinfection studies (Fig. 6A). For re-activation, the rested mice were treated with immunosuppressive drug dexamethasone for 30 days (Fig. 6A).

A synergistic effect in the bacterial reduction was observed in the infected mice treated with BPM along with INH/RIF, indicating that BPM co-treatment extensively enhances the anti-tubercular potential of present available antituberculosis therapy (Fig. 6B and C).

The INH/RIF/BPM treated and reinfected mice displayed increased anti-tubercular immunity as evidenced by a significant reduction in the bacterial burden in the lungs (Fig. 6D) and the spleen (Fig. 6E) of these mice compared to the group that received only INH/RIF. BPM co-treatment also reduced the TB reactivation rate from 70% to 40% (Fig. 6F). Overall, these results signify the potential of BPM as an adjunct immunomodulator for TB therapy.

## DISCUSSION

The current regime for TB treatment has been proven to dampen the host’s immune system, which allows partial reduction of bacterial burden in the infected individuals (39). This further leads to the emergence of drug-resistant TB, such as MDR, XDR, and now recently emerging TDR (40). Therefore, researchers are now pacing toward immunomodulatory therapy that can efficiently control mycobacterial infection.

BPM is a wide-spectrum anti-bacterial drug that has been shown to impart substantial immunity against drug-susceptible TB in the *in vitro* and *in vivo* mice models. It acts synergistically with rifampicin and thereby confers better protection against *M.tb* (12). In this study, we have tried to evaluate the effect of BPM on the host immune system and understand the mechanism of action of BPM in the reduction of *M.tb* load.

Innate immune cells like macrophages are the first line of defense against *M.tb*. These immune cells subsequently help prime the adaptive immune cells like T cells to improve the disease outcome and provide long-term immunity (41). ROS is one of the well-studied features employed by macrophages to combat intracellular pathogens by activating a plethora of signaling pathways that ultimately result in the generation of a pro-inflammatory milieu for pathogen elimination (42). Consistent with this understanding, BPM treatment in peritoneal macrophages significantly enhanced ROS generation and activation of the p38 signaling pathway, which is known to induce pro-inflammatory cytokines like IL-1β. It has been reported that IL-1β from innate cells like macrophages and the dendritic cell can prime T cells and result in their activation and differentiation (43). Various studies and models have postulated that the balance between pro-inflammatory and anti-inflammatory cytokines is necessary to culminate the bacterial progression in infected individuals (44). Assorted reports suggest that IFNγ producing T cells provide immunity against TB, and thereby T cells priming is essential to impart immunity against the bacteria (45).

To substantiate our encouraging *ex vivo* findings, we further assessed the impact of BPM on the modulation of the host immune system. We observed that upon BPM treatment, the innate cells expressing co-stimulatory and activation markers, such as CD80, CD86, and CD40, were enhanced at the primary site of infection, i.e., lungs. These observations correlate that upon *M.tb* infection, BPM enhances the activation of dendritic cells and macrophages to increase the antigen presentation to the T cells leading to enhanced T cell activation. Indeed, BPM treatment increased the activation of CD4^+^ and CD8^+^ T cells in the lungs and the spleen of the *M.tb* infected mice as evidenced by the increase in the expression of CD69. Further, these T cells demonstrated enhanced production of the pro-inflammatory cytokine, IFN-γ, which is known to have a protective role against *M.tb* (46). Anther pro-inflammatory cytokine, IL-17, which is known to impart long-term immunity (47), was significantly increased in the BPM treated mice.

Classically, it has been acknowledged that long-term memory plays a crucial role in protection against secondary infection with *M.tb*. Upon antigen encounter, Naïve T cells differentiate into central memory T cells (T_CM_), which are further differentiated in secondary lymphoid organs into effector memory cells (T_EM_) (48, 49). Therefore, we evaluated the potential of BPM in modulating T cell memory responses to mediate long-term memory against *M.tb*. Recently we and others have shown how antimycobacterial compounds elicit *M.tb* specific long-term immunity (50–52). Here, again, we observed that BPM boosted the memory responses in both the lung and the spleen of infected animals.

The anti-tubercular drugs are known to have an immune dampening effect and fail to provide complete sterility. Therefore, we evaluated BPM as an adjunct therapy with DOTS and observed that BPM co-administration significantly enhanced the anti-tubercular potential of current front-line anti-TB drugs, INH, and RIF. This was also supported by our findings that the extent of TB recurrence due to reinfection and reactivation was also reduced significantly with BPM as an adjunct.

**FIG 7:**
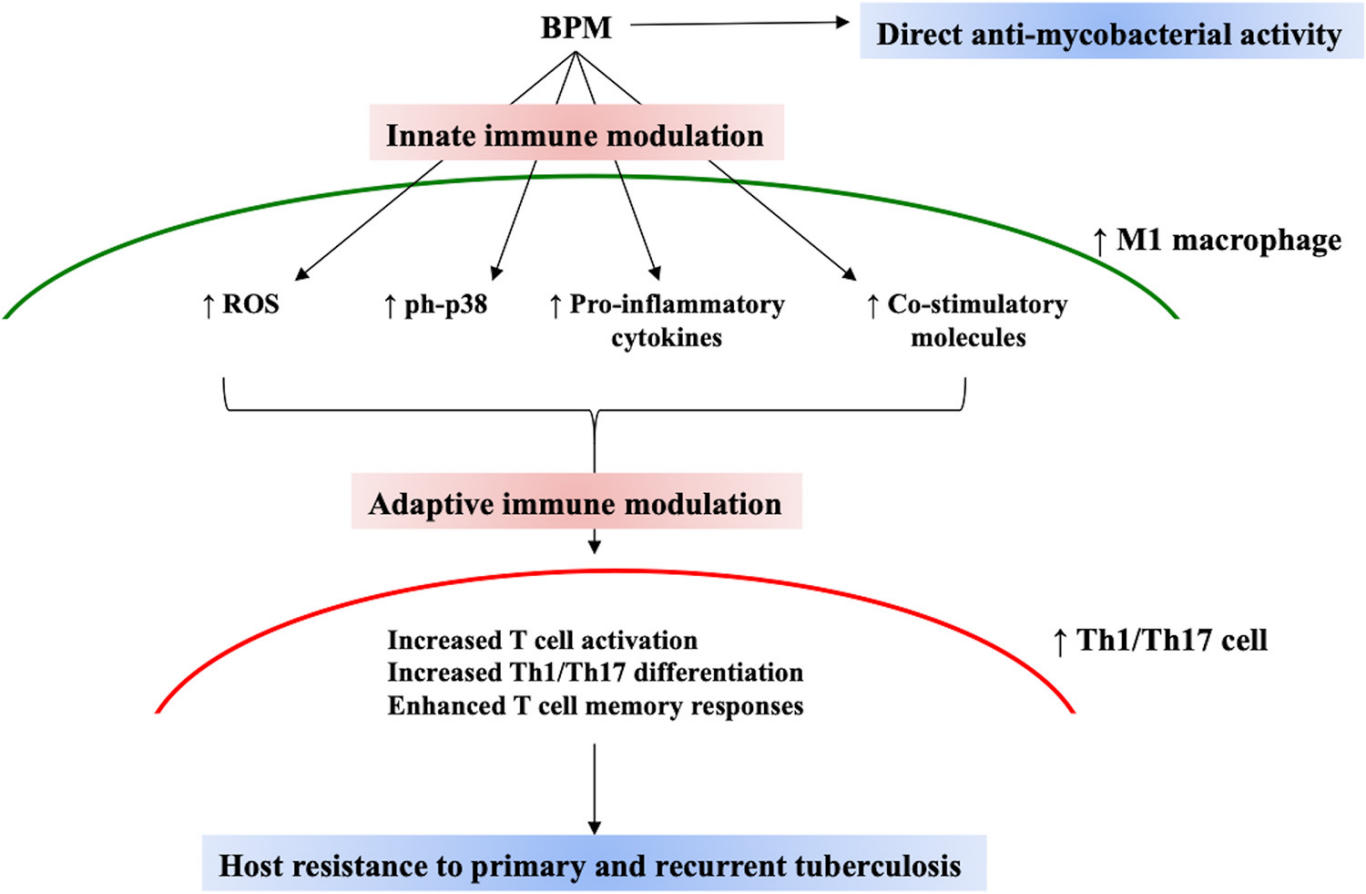
Proposed mechanism of action of BPM during *M.tb* infection. As a carbapenem, BPM has direct anti-mycobacterial effect. BPM also exerts immunomodulatory effects on the innate and adaptive immune arm of the host. In macrophages, BPM treatment leads to increased levels of ROS, p38 activation, pro-inflammatory cytokine secretion, and co-stimulation indicative of M1 macrophage phenotype. This further leads to positive modulation of T cells in terms of activation and Th1/Th17 environment, which is beneficial for the host.

Apart from its classical role as an anti-tubercular drug, our findings suggest that BPM also functions as an immunomodulator. Collectively, it can be proposed that BPM possesses broad-spectrum activities and thus can be projected as a potential adjunct immunotherapeutic against TB (Fig. 7).

## MATERIALS AND METHODS

### Ethics statement.

Mice were procured from the ICGEB animal facility and the experiments were performed as per the instructions specified by the Institutional Animal Ethics Committee of the International Centre for Genetic Engineering and Biotechnology (ICGEB, New Delhi, India) together with the Department of Biotechnology (DBT) standards (Government of India) (Approval ID: ICGEB/IAEC/18092021/IMB-19). The animals utilized were virtuously sacrificed by asphyxiation with carbon dioxide in accordance with institutional and DBT practices.

### Mice.

C57BL/6 mice of 6 to 8 weeks were nurtured and maintained in the Animal facility at ICGEB, New Delhi, India. Mice were procured for the experimental strategy from the facility.

### Bacterial strains.

Mid log phase cultures of *Mtb* (H37Rv and Rv-GFP), MDR (JAL 2261) and XDR (MYC 431) strains were used for the study. For culturing of the mycobacterial strains 7H9 (Middlebrooks, Difco, 462) medium supplemented with 10% OADC (Oleic acid, albumin, dextrose, and catalase; 463 Difco), 0.05% Tween 80, and 0.2% glycerol were used. Bacterial stocks prepared in 20% glycerol were cryopreserved at −80°C, and these stocks were further used for all the infection experiments in the study.

### Isolation of mouse peritoneal macrophages.

C57/BL6 mice aged 6 to –8 weeks were administered intraperitoneally with sterile thioglycolate (2 mL; Brewer modified, BBL, BD Biosciences: 4% wt/vol in water). Post 5 days, macrophages were isolated from the peritoneal lavage by flushing them with ice-chilled PBS. The cells were pelleted down and resuspended in RPMI 1640 media supplemented with 10% heat-inactivated fetal calf serum and 1% antibiotic (Penicliin-Streptomycin). Cell viability was determined by trypan blue dye staining using a hemocytometer. Cells were seeded and incubated overnight at 37°C and 5% CO_2_. Non-adherent cells were washed off using sterile 1X PBS, and the adherent macrophage monolayer was further infected with 1:1 MOI of Rv-GFP and 1:10 MOI of H37Rv.

### *Ex vivo* infection and CellROX assay.

Mouse peritoneal macrophages were used for *ex vivo* experimental procedures. Mid log phase cultures were maintained from bacterial cryostocks. Single cell suspension of the bacterial cultures was prepared by passing it through a 23 ½ gauge syringe 10, followed by a 26 ½-gauge syringe. Macrophages were infected with H37Rv and H37Rv-GFP at MOI 1:10 and 1:1, respectively. Post 4 h of infection, cells were washed twice with 1X PBS to terminate *M.tb* infection. The cells were then treated with 10 mM NAC or 10 μg/mL BPM and incubated at 37°C and then analyzed for intracellular ROS and bacterial burden. CellROX Deep Red Reagent (ThermoFisher Scientific) was used to estimate the ROS levels as per the protocol supplied by the manufacturer.

### *In vivo* mice infection and CFU determination.

Mice were infected via aerosol route with a drug- susceptible strain of *M.tb* (H37Rv) to deliver 110 viable bacilli to mouse lungs. Madison aerosol chamber (University of Wisconsin) with its pre-adjusted nebulizer containing 15 mL of bacterial single cell suspension was used for infection. Mice from each group were sacrificed at indicated time points to determine the bacterial load. For CFU enumeration, harvested lung and spleen from designated groups of mice were homogenized in sterile 1X PBS and then plated on 7H11 Middlebrooks (Difco) plates containing 0.05% Tween 80, 10% oleic acid, albumin, dextrose, and catalase (OADC) (Difco) in different dilutions. The plates were then incubated at 37°C and the colonies were examined after 3 weeks.

### Drug delivery.

For *ex vivo* experiments, cells were treated with 10 μg/mL of BPM (Sigma-Aldrich). Furthermore, for long-term mice studies, 5 mg/kg of BPM dissolved in water was administered intraperitoneally for 45 days thrice a week, and the control group received only the vehicle. A total of 100 mg/L of INH and 60 mg/L RIF was given in drinking water which was replaced on alternative days.

### Protein isolation and Western blotting.

Peritoneal macrophages seeded at a density of 1 × 10^6^cells/mL were infected with H37Rv at an MOI of 1:10. After 2 h of infection, cells were washed twice with 1X PBS to terminate the *M.tb* infection. The infected cells or uninfected cells were treated with or without 10uM of BPM for 2 h. Post 2 h of treatment, RIPA lysis buffer (50 mM Tris, pH 8.0, 150 mM NaCl, 1.0% NP-40, 0.5% Sodium deoxycholate, and 0.1% SDS) containing 1X protease inhibitor cocktail was used to prepare whole-cell lysates. Post centrifugation of lysate at 13,000 × *g*, the aqueous layer was removed and quantified for protein using Bradford reagent. Protein samples were electrophoresed using 10% polyacrylamide gel (SDS-PAGE) and electroblotted on a nitrocellulose membrane. The nitrocellulose membrane was blocked using 5% BSA solution in PBST (PBS and 0.1% Tween 20) for 2 h. After blocking, the membrane was probed with respective antibodies from CST overnight at 4°C. Blots were developed using Chemiluminescent HRP substrate (ECL, Millipore) and visualized on ImageQuant LAS 500.

### RNA isolation and qPCR.

Cells were lysed using TRIzol reagent and whole RNA was extracted by the standard RNA isolation protocol. The cDNA was synthesized from isolated RNA using the iScript cDNA synthesis kit. Bio-Rad Real-time thermal cycler was further used to set up the reactions using SYBR green Master (Bio-Rad) according to the manufacturer’s protocol. The primer sequences used in the study are given in Table 1.

**Table 1:** The primer sequences used in the study

Primer	Sequence (5′–3′)
IL-10 Forward Primer	CATGGGTCTTGGGAAGAGAA
IL-10 Reverse Primer	AACTGGCCACAGTTTTCAGG
IL-12β Forward Primer	AAGGAACAGTGGGTGTCCAG
IL-12β Reverse Primer	GGAGACACCAGCAAAACGAT
IL-1β Forward Primer	CCCAAGCAATACCCAAAGAA
IL-1β Reverse Primer	GCTTGTGCTCTGCTTGTGAG
IL-22 Forward Primer	ATGAGTTTTTCCCTTATGGGGAC
IL-22 Reverse Primer	GCTGGAAGTTGGACACCTCAA
IL-23 Forward Primer	AATAATGTGCCCCGTATCCA
IL-23 Reverse Primer	AGGCTCCCCTTTGAAGATGT
IL-6 Forward Primer	CCGGAGAGGAGACTTCACAG
IL-6 Reverse Primer	TCCACGATTTCCCAGAGAAC
TNF-α Forward Primer	TAGCCAGGAGGGAGAACAGA
TNF-α Reverse Primer	TTTTCTGGAGGGAGATGTGG
β-Actin Forward Primer	GCTGGAAGTTGGACACCTCAA
β-Actin Reverse Primer	CCAGTTGGTAACAATGCCATGT

### Reinfection and reactivation assessment.

To determine the vulnerability of *M.tb* infection, mice models were infected with low dose aerosol infection of the H37Rv strain. This was followed by INH (0.1g/L) and/RIF (0.06g/L) treatment for 60 days followed by 30 days’ rest. During reactivation experiments, mice were given dexamethasone (5 mg/kg) intraperitoneally, thrice a week for 30 days followed by euthanizing at indicated time points to examine immunological response and bacterial burden. For reinfection studies, another group was challenged with *M.tb.* Post 30 days of infection, the mice were sacrificed to determine the bacterial burden.

### Flow cytometry.

Single cell suspension of lung and spleen were prepared from mice of different groups by macerating using frosted slides in ice-cold RPMI 1640 (HyClone) supplemented with 10% FBS. For comprehensible analysis of isolated cell population RBCs were further lysed by RBC lysis buffer with 10% RPMI 1640. A total of 1 × 10^6^ cells per well were seeded in 12-well plates for staining experiment. For surface staining, cells were *ex vivo* stimulated with 10 μg/mL of H37Rv complete soluble antigen (CSA). Eventually, 0.5 μg/mL Brefeldin A and 0.5 μg/mL of Monensin solution (BioLegend) were added prior to the last 4 h of culture at 37°C and 5% CO_2_. After washing the cells twice with FACS buffer (PBS + 3% FBS) and staining with the respective to the surface markers, cell fixation was done with 100 μL fixation buffer (Biolegend) for 30 min. For intracellular staining, 1X permeabilizing buffer (Biolegend) was used to internalize the antibodies and then stained with fluorescently labeled anti-cytokine antibodies. For non-fluorochrome labeled antibodies, secondary antibody tagged with Alexa Fluor 488 was employed. Acquisition was completed by flow cytometry (BD LSRFortessa Cell Analyzer - Flow Cytometers, BD Biosciences) followed by data analysis via FlowJo (Tree Star).

### Antibodies.

Antibodies used in the studies are mentioned below:

Anti-Mouse: CD3-Pacific Blue, CD4-PE, CD8-APCCy7, CD69-FITC, CD44-FITC, CD62L-APC, IFNγ-APC, IL-17-PECy7, CD11b-APCCy7, CD11c-APC, CD80-FITC, CD86-PerCPCy5.5, CD40-PE, CD4-APC, and CD4-FITC from Biolegend, USA.

Anti-mouse: p38, ph-p38 and β-Actin from Cell Signaling Technology.

### Statistical analysis.

GraphPad Prism Software was used to evaluate the executed experimental data and the significant variation among the group was analyzed by 2 tailed unpaired Student's *t* test or 1-way ANOVA. *, *P* < 0.05, **, *P* < 0.005, ***, *P* < 0.0005.

### Data availability.

All data are contained within the manuscript.
