# Supplementary material for: Biapenem, a Carbapenem Antibiotic, Elicits Mycobacteria Specific Immune Responses and Reduces the Recurrence of Tuberculosis
Source: Microbiol Spectr. 2023 Jun 5;11(4):e00858-23. doi: 10.1128/spectrum.00858-23 (PMC10434282; doi:10.1128/spectrum.00858-23)
Supplement: Supplemental file 1 — Supplemental material. Download spectrum.00858-23-s0001.docx, DOCX file, 0.2 MB [file spectrum.00858-23-s0001.docx]

**Supplementary Figure and Legend:**


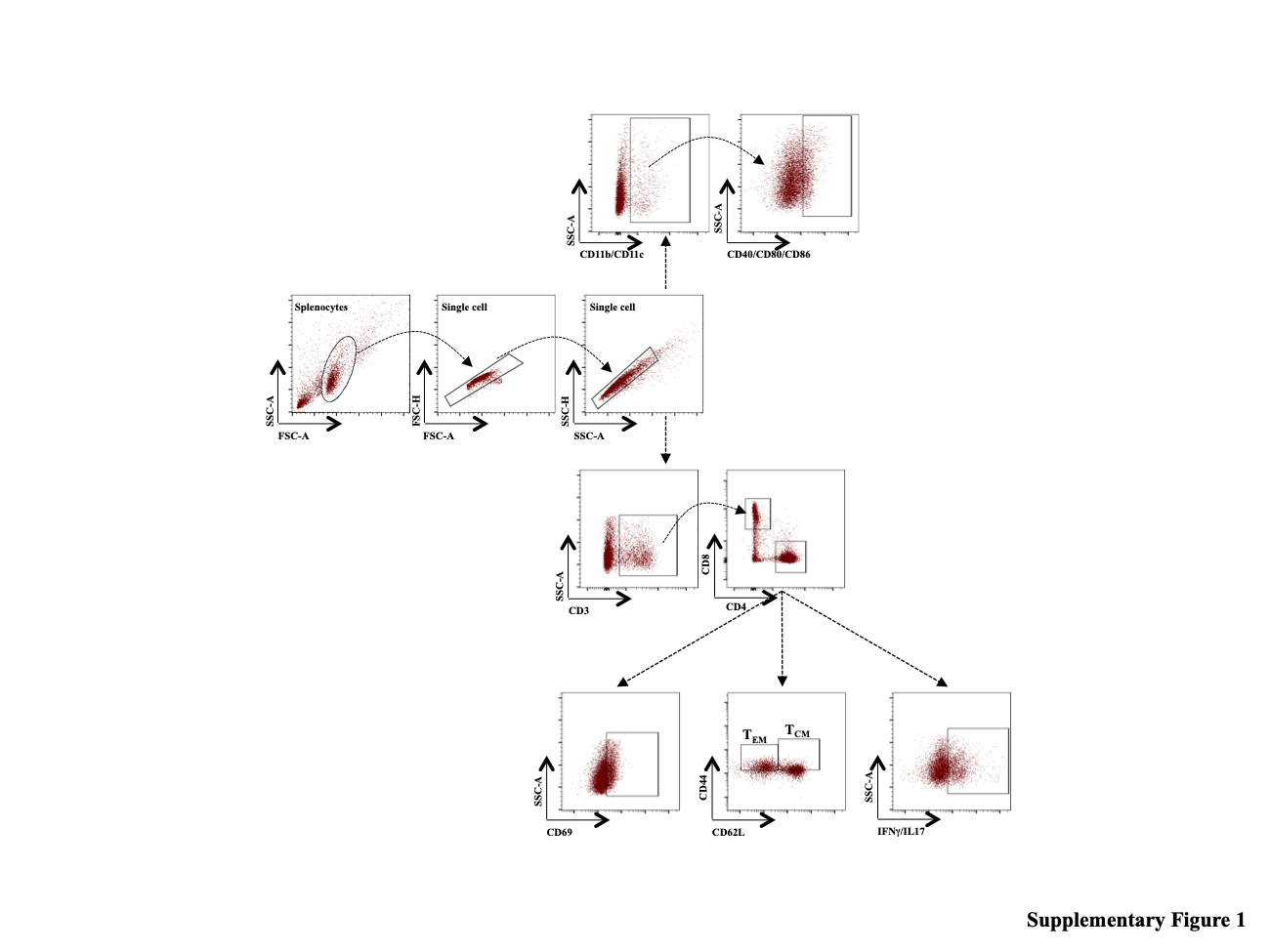


**Supplementary Figure 1: Gating strategy employed to profile immune cells.**
